# Cov19VaxKB: A web-based integrative COVID-19 vaccine knowledge base

**DOI:** 10.1016/j.jvacx.2021.100139

**Published:** 2021-12-28

**Authors:** Philip C. Huang, Rohit Goru, Anthony Huffman, Asiyah Yu Lin, Michael F. Cooke, Yongqun He

**Affiliations:** a College of Literature, Science, and the Arts, University of Michigan, Ann Arbor, MI 48109, USA; b Department of Computational Medicine and Bioinformatics, University of Michigan, Ann Arbor, MI 48109, USA; c National Human Genome Research Institute, National Institutes of Health, Bethesda, MD 20892, USA; d School of Information, University of Michigan, Ann Arbor, MI 48109, USA; e Unit for Laboratory Animal Medicine, University of Michigan Medical School, Ann Arbor, MI 48109, USA; f Department of Microbiology and Immunology, University of Michigan Medical School, Ann Arbor, MI 48109, USA

**Keywords:** Vaccine, SARS-CoV-2, COVID-19, COVID-19 vaccine, Database, Knowledge base, Bioinformatics, Adverse event, VAERS, Cov19VaxKB, Ontology, AE, adverse event, CDC, Centers for Disease Control and Prevention, COVID-19, Coronavirus disease 2019, FDA, Food and Drug Administration, MERS-CoV, Middle Eastern Respiratory Syndrome, NCBI, National Center for Biotechnology Information, PMID, PubMed identification number, PRR, Proportional Reporting Ratio, OWL, Web Ontology Language, SARS-CoV, Severe Acute Respiratory Syndrome Coronavirus, SARS-CoV-2, Severe Acute Respiratory Syndrome Coronavirus 2, VAERS, Vaccine Adverse Event Reporting System, VIOLIN, Vaccine Investigation and Online Information Network, VO, Vaccine Ontology, WHO, World Health Organization

## Abstract

The development of SARS-CoV-2 vaccines during the COVID-19 pandemic has prompted the emergence of COVID-19 vaccine data. Timely access to COVID-19 vaccine information is crucial to researchers and public. To support more comprehensive annotation, integration, and analysis of COVID-19 vaccine information, we have developed Cov19VaxKB, a knowledge-focused COVID-19 vaccine database (http://www.violinet.org/cov19vaxkb/). Cov19VaxKB features comprehensive lists of COVID-19 vaccines, vaccine formulations, clinical trials, publications, news articles, and vaccine adverse event case reports. A web-based query interface enables comparison of product information and host responses among various vaccines. The knowledge base also includes a vaccine design tool for predicting vaccine targets and a statistical analysis tool that identifies enriched adverse events for FDA-authorized COVID-19 vaccines based on VAERS case report data. To support data exchange, Cov19VaxKB is synchronized with Vaccine Ontology and the Vaccine Investigation and Online Information Network (VIOLIN) database. The data integration and analytical features of Cov19VaxKB can facilitate vaccine research and development while also serving as a useful reference for the public.

## 1 Introduction

The emergence of coronavirus disease 2019 (COVID-19), caused by the Severe Acute Respiratory Syndrome Coronavirus 2 (SARS-CoV-2), has severely impacted human populations on a global scale. As of November 8, 2021, over 246 million confirmed cases of COVID-19 had been recorded worldwide since the start of the COVID-19 pandemic, resulting in nearly 5 million deaths [1]. To reduce the transmission of SARS-CoV-2 within human populations, researchers worldwide have developed vaccines immunizing against SARS-CoV-2. Several vaccines, including Pfizer-BioNTech’s Comirnaty, Moderna’s mRNA-1273, and Oxford-AstraZeneca’s AZD1222, have been authorized for public use in at least one country, while many other vaccines are currently undergoing preclinical studies or Phase 1–3 clinical trials. These recent developments have generated an influx of new information about the composition, production, distribution, and effects of COVID-19 vaccines.

Many online resources on COVID-19 vaccine information currently exist, providing product-related information such as vaccine type, antigen, storage, adjuvant, and research status as well as host-related data regarding vaccine efficacy, immunogenicity, and safety. For instance, extensive clinical trial data for COVID-19 vaccines can be found on the website clinicaltrials.gov and other clinical trial record websites operated by governmental agencies. The CDC and FDA’s Vaccine Adverse Event Reporting System (VAERS) contains a detailed repository of COVID-19 vaccine adverse event information (https://vaers.hhs.gov/). As of November 8, 2021, PubMed contained 21,720 publications related to COVID-19 vaccines using the search query “(vaccine OR vaccination) AND (SARS-CoV-2 OR COVID-19)”. There are also several online COVID-19 vaccine trackers that provide an overview of vaccines that are undergoing development or have been authorized for public use, including the World Health Organization’s COVID-19 Vaccine Tracker and Landscape [2], the London School of Hygiene and Tropical Medicine VaC tracker [3], and the New York Times Coronavirus Vaccine Tracker [4]. However, these resources typically focus on one or a few of these vaccine-related topics for a specific group of users, such as adverse events, clinical trials, or general vaccine or vaccination information. As more relevant data is generated, an organized, accessible knowledge base that integrates COVID-19 vaccine information from various sources is necessary.

Existing data curation, integration, and analysis systems that focus on vaccine information include the Vaccine Investigation and Online Information Network (VIOLIN) database and Vaccine Ontology. VIOLIN is a web-based, publicly accessible vaccine database that includes information about over 4000 vaccines for over 200 pathogens and non-infectious diseases (http://www.violinet.org) [5]. VIOLIN also includes many small databases and features such as the Vaxign2 vaccine design program [6] and VO-SciMiner, an ontology-based literature mining tool [7]. Vaccine Ontology (VO) is a community-based ontology that covers different aspects of vaccines and vaccination, including vaccine components, formulations, and host responses [8], [9].

To address the need for a publicly accessible and integrated repository of COVID-19 vaccine information, we have developed the COVID-19 Vaccine Knowledge Base (Cov19VaxKB). Developed as a relatively independent program under the umbrella of the VIOLIN system, Cov19VaxKB is focused on the collection, annotation, and integration of COVID-19 vaccine information encompassing vaccine development, production, safety, immunogenicity, efficacy, and more. Cov19VaxKB also contains features that allow users to analyze data related to vaccine efficacy, safety, and mechanisms. The knowledge base is freely available for public use and can be accessed at http://www.violinet.org/Cov19VaxKB.

## 2 Methods

### 2.1 Cov19VaxKB system and database design

**Fig. 1:**
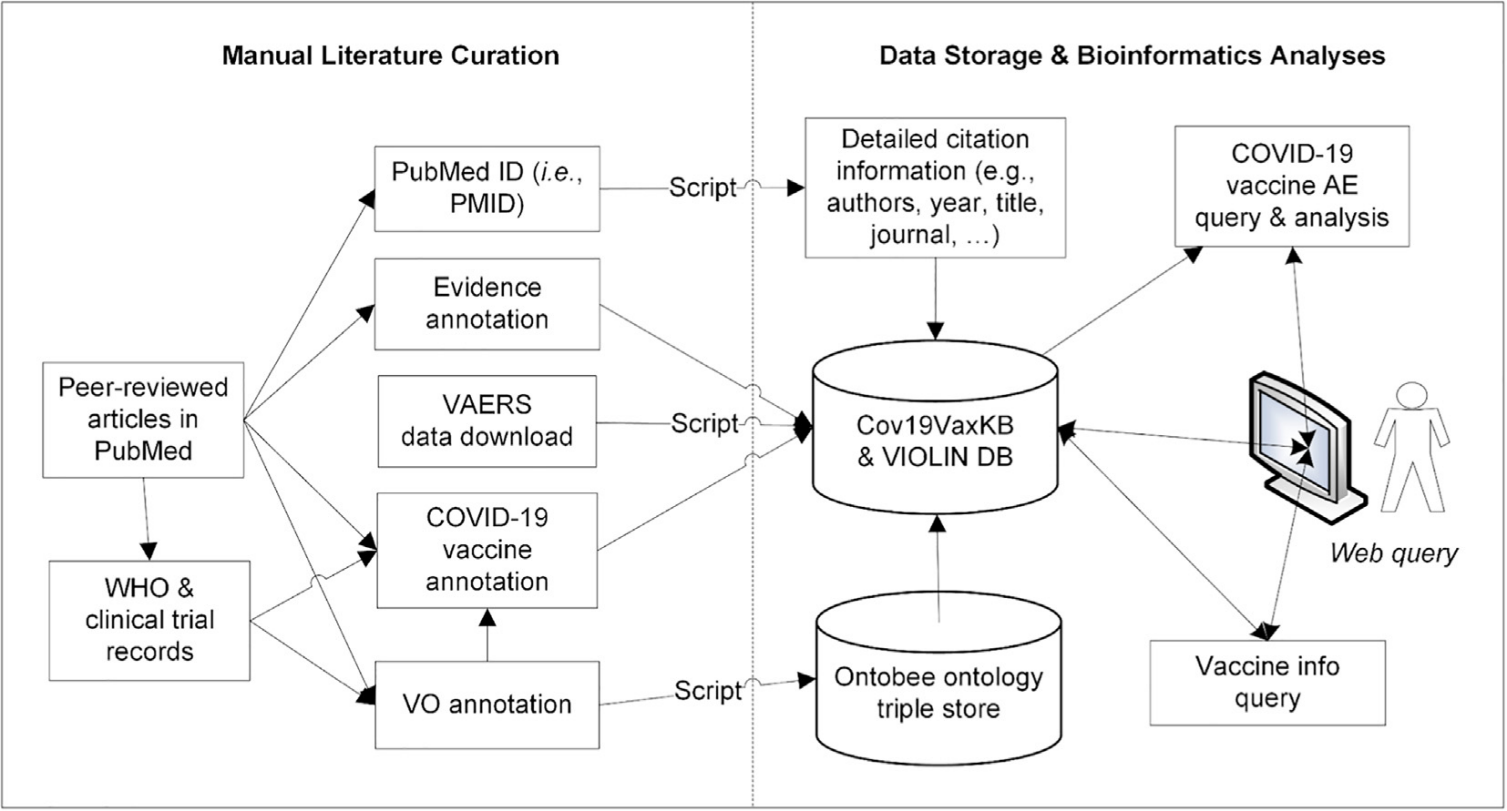
Cov19VaxKB workflow and system design. COVID-19 vaccine data from peer-reviewed PubMed publications, clinical trial records, and the WHO “Draft landscape and tracker of COVID-19 candidate vaccines” was annotated and stored into the Cov19VaxKB and VIOLIN databases. Data sharing and transfer was enabled by Vaccine Ontology (VO) IDs assigned for COVID-19 vaccines. Adverse event case report data was extracted from VAERS and analyzed in Cov19VaxKB using a server-side script.

Cov19VaxKB was established within the VIOLIN database system using two virtual servers in the University of Michigan Medical School virtual server system that runs the Redhat Enterprise Linux operating system [5]. It is developed with classical three-tier architecture. The knowledge base website features a series of comprehensive vaccine lists, a vaccine adverse event analysis program, a vaccine design tool, an automated literature search feature, a list of vaccine news updates, and links to other COVID-19 vaccine resources. Fig. 1 illustrates the workflow of the Cov19VaxKB/VIOLIN database design and implementation.

### 2.2 Annotation of vaccine information

Data in Cov19VaxKB is manually curated and annotated through two platforms: the knowledge base’s vaccine list web pages and the VIOLIN web-based data curation system.

The vaccine list pages are constructed using the PHP programming language. Data within the vaccine lists is primarily derived from the WHO’s “COVID-19 Vaccine Tracker and Landscape,” which contains an extensive list of all COVID-19 vaccines in all stages of development as well as their associated clinical trial IDs (https://www.who.int/publications/m/item/draft-landscape-of-covid-19-candidate-vaccines). This resource is used to gather information about vaccine names, vaccine type, manufacturer, route of administration, number of doses, length of time between doses, and clinical trial IDs. In addition, clinical trial record URLs, age subgroups, and location are derived from clinical trial websites such as clinicaltrials.gov. Relevant publications are identified by searching the name of the vaccine of interest on PubMed. Links to corresponding VIOLIN and Vaccine Ontology entries are also incorporated into these lists. When applicable, the date on which a vaccine was first authorized by a regulatory agency is sourced from a manual web search. All information from these resources is manually curated into PHP files, which are then uploaded to the Cov19VaxKB server for display. The vaccine list pages are organized according to vaccine development status, including preclinical studies, Phase 1–3 clinical trials, and authorization for emergency or full use. These lists are updated weekly to ensure that the information provided is up-to-date and accurate.

The VIOLIN data curation system is also utilized for manual curation of data in Cov19VaxKB [5]. VIOLIN entries for each vaccine contain product information, such as manufacturer, vaccine type, antigen, and immunization route, as well as host response data from preclinical and clinical studies, including vaccine efficacy, immune response, and side effects. These entries can be accessed through the Cov19VaxKB query feature described in the next section.

### 2.3 Cov19VaxKB data query and result display

The Cov19VaxKB web interface includes a query for COVID-19 vaccine entries that are stored in the VIOLIN database. The query is submitted from the Cov19VaxKB web user interface (the presentation tier) and is then processed using PHP/SQL (the middle tier, application server) against a MySQL relational database (the data tier, database server). Query results are then displayed in an accessible web browser.

### 2.4 Cov19VaxKB vaccine adverse event data analysis tool

The vaccine adverse event analysis tool in Cov19VaxKB contains a query for adverse event case report information derived from VAERS and a statistical analysis feature. Case report data for all vaccines is downloaded monthly from the CDC VAERS database and deposited into a local MySQL database. Through a server-side script, the data is parsed and filtered for COVID-19 vaccines. The resulting case report data is then formatted to include attributes such as vaccine name, USA state or territory, age and sex of vaccine recipient, year of vaccination, and VAERS report year. This formatting allows users to query and filter adverse events for a specific COVID-19 vaccine based on the attributes described above. Users can also select a specific adverse event to access comprehensive tables of individual VAERS case reports.

To display a potential association between a specific adverse event (AE) and a COVID-19 vaccine, three statistical measures are calculated: a Chi-squared value with its associated degrees of freedom and p-value, Proportional Reporting Ratio (PRR) [10], and case report frequency [11]. An R script for a Pearson Chi-square test with Yates’ continuity correction uses a 2 × 2 frequency/contingency table to calculate the Chi-squared value, degrees of freedom, and p-value. The PRR represents the frequency of an adverse event for a vaccine of interest relative to all other case reports for all vaccines in the VAERS database. To determine whether a specific AE is significantly enriched for a specified COVID-19 vaccine, we have used a set of significance cutoffs as reported previously [11], which includes three criteria: Chi-squared value > 4, PRR > 2, and number of case reports > 0.2% of total case reports for the specified vaccine. All three criteria need to be met to identify the AE as significantly enriched for the vaccine.

Using the Cov19VaxKB statistical analysis tool and cutoff criteria, we generated a list of statistically significant adverse events for the Pfizer-BioNTech, Moderna, and Johnson & Johnson (Janssen) vaccines. These adverse events were systematically compared and analyzed among the three vaccines.

### 2.5 Cov19VaxKB vaccine automated literature update tool

Cov19VaxKB features an automated literature update tool that lists COVID-19 vaccine-related publications that have been published within the current and previous months. Publications are extracted from PubMed via NCBI’s E-utilities data retrieval program and are formatted and displayed in HTML webpages using a PHP script [12]. One HTML webpage features 100 SARS-CoV-2/COVID-19 vaccine publications and 100 coronavirus vaccine publications published during the current month that were extracted from PubMed. A second HTML webpage displays 100 SARS-CoV-2/COVID-19 vaccine publications and 100 coronavirus vaccine publications from PubMed that were published in the previous month. The queries for the SARS-CoV-2/COVID-19 and coronavirus vaccine publication lists use the keywords “(vaccine OR vaccination) AND (SARS-CoV-2 OR COVID-19)” and “(vaccine OR vaccination) AND (Coronavirus),” respectively. In all queries, the results are filtered by date. To update the publication lists automatically, the PHP script is run daily using a server-side cron job [13]. Direct links to PubMed queries of COVID-19 vaccine and coronavirus vaccine publications are also included.

### 2.6 Cov19VaxKB vaccine design tool

We have previously developed a web application for vaccine design (Vaxign2), which utilizes reverse vaccinology and machine learning to predict vaccine targets [6]. The Cov19VaxKB version of this vaccine design tool includes an embedded view of the SARS-CoV-2 results from Vaxign2. The SARS-CoV-2 Vaxign2 output includes the protein name and accession number, adhesin probability, number of trans-membrane helices, and a Vaxign-ML score derived from a machine learning-based prediction [14].

### 2.7 Cov19VaxKB data transfer and download

To enable data transfer, Cov19VaxKB is synchronized with VO, which serves as an ontological storage system for information regarding vaccine names, vaccine type, route of administration, manufacturers, antigens, host species, and adjuvants [9]. VO entries for COVID-19 vaccines are manually created and updated using the Protégé ontology program in the Web Ontology Language (OWL). Links to these VO entries are manually incorporated into the knowledge base’s vaccine lists and VIOLIN entries. Excel files of the vaccine lists, Vaxign2 output, and adverse event analysis results are uploaded to the “Data Download” webpage for user download.

## 3 Results

### 3.1 Overall Cov19VaxKB system design and statistics

The Cov19VaxKB system is designed to focus on three aspects of COVID-19 vaccine data: vaccine development, product-side information, and host-side information.

Vaccine development information includes data about clinical trials and pre-clinical research studies of newly developed COVID-19 vaccines. Product-side information refers to vaccine type, antigens, adjuvants, manufacturer, and storage. Host-side data includes information regarding immune responses, efficacy, and adverse events.

As of November 6, 2021, Cov19VaxKB vaccine lists stored a total of 315 COVID-19 vaccines that are in preclinical studies, Phase 1–3 clinical trials, or are authorized for emergency or full use. Out of 315, there were 194 preclinical vaccines, which are undergoing non-clinical research studies. There are 34 Phase I vaccines and 40 Phase II vaccines, which are being evaluated for safety and immunogenicity. Currently, there are 24 Phase III vaccines, which are being assessed for safety and efficacy, and 23 vaccines that are authorized for emergency or full use in at least one country. Table 1 contains a comprehensive list of all authorized COVID-19 vaccines with manufacturer information, authorization date, number of doses, timing of doses, and VIOLIN and VO ID’s, while Supplemental File 1 lists all unauthorized COVID-19 vaccines in Phase 1–3 clinical trials.

**Table 1:** List of COVID-19 vaccines authorized for emergency or full use as of November 8, 2021. Manufacturer information, date of first authorization, number of doses, timing of doses, and corresponding VIOLIN and VO entries are also listed.

**Vaccine Name**	**Manufacturer**	**Date of First Authorization**	**Number of Doses**	**Timing of Doses**	**VIOLIN ID**	**VO ID**
**DNA Vaccines**						
ZyCoV-D	Zydus Cadila	August 20, 2021	3	Day 0 + 28 + 56	5778	VO_0005162

BBIBP-CorV	Beijing Institute of Biological Products; Sinopharm	July 2020	2	Day 0 + 21	5776	VO_0005166
Chinese Academy of Medical Sciences COVID-19 vaccine (Covidful)	Institute of Medical Biology, Chinese Academy of Medical Sciences	June 9, 2021	2	Day 0 + 28	5785	VO_0005167
CoronaVac (PiCoVacc)	Sinovac	August 28, 2020	2	Day 0 + 14	5761	VO_0005141
COVAXIN (BBV152)	Bharat Biotech	January 3, 2021	2	Day 0 + 14	5795	VO_0004991
COVIran Barakat	Shifa Pharmed Industrial Co	June 13, 2021	2	Day 0 + 14	5815	VO_0005229
CoviVac	Chumakov Centre at the Russian Academy of Sciences	February 20, 2021	2	Day 0 + 14	5852	VO_0005243
KCONVAC	Shenzhen Kangtai Biological Products Co., Ltd., Beijing Minhai Biotechnology Co.	May 14, 2021	2	Day 0 + 28	5805	VO_0005084
QazVac (QazCovid-in)	Research Institute for Biological Safety Problems, National Scientific Center for Phthisiopulmonology of the Republic of Kazakhstan	January 13, 2021	2	Day 0 + 21	5810	VO_0005093
WIBP-CorV	Wuhan Institute of Biological Products/ Sinopharm	February 25, 2021	2	Day 0 + 21	5775	VO_0005160

**RNA Vaccines**						
Comirnaty (BNT162b2 , Tozinameran)	BioNTech/Fosun Pharma/Pfizer	December 11, 2020	2	Day 0 + 21	5784	VO_0004987
mRNA-1273	Moderna	December 18, 2020	2	Day 0 + 28	5789	VO_0005157

**Recombinant Vector Vaccines**						
Ad26.COV2.S (JNJ-78436735)	Janssen Pharmaceutica	February 27, 2021	1–2	Day 0 or Day 0 + 56	5782	VO_0005159
Ad5-nCoV (Convidicea)	CanSino Biologics	June 25, 2020	1	Day 0	5768	VO_0005144
AZD1222 (ChAdOx1 nCoV19, Covishield)	AstraZeneca/University of Oxford	December 30, 2020	2	Day 0 + 28	5774	VO_0005158
Sputnik V (Gam-COVID-Vac)	Gamaleya Research Institute	August 11, 2020	2	Day 0 + 21	5777	VO_0005163

**Subunit Vaccines**						
Abdala	Center for Genetic Engineering and Biotechnology (CIGB)	May 12, 2021	3	Day 0 + 14 + 28 or Day 0 + 28 + 56	5803	VO_0005082
COVAX-19 (SpikoGen)	Vaxine, CinnaGen	October 6, 2021	2	Day 0 + 21	5829	VO_0005193
EpiVacCorona	FBRI State Research Center of Virology and Biotechnology	October 14, 2020	2	Day 0 + 21	5794	VO_0005088
MVC-COV1901	Medigen, Dynavax	July 19, 2021	2	Day 0 + 28	5828	VO_0005192
NVX-CoV2373 (Covovax)	Novavax	November 1, 2021	2	Day 0 + 21	5791	VO_0005155
Soberana 02	Instituto Finlay de Vacunas	May 12, 2021	2	Day 0 + 28	5814	VO_0005097
ZF2001	Anhui Zhifei Longcom Biopharmaceutical; Institute of Microbiology, Chinese Academy of Sciences	March 1, 2021	2–3	Day 0 + 28 or Day 0 + 28 + 56	5764	VO_0005142

The literature update tool automatically extracts PubMed citations related to SARS-CoV-2/COVID-19 vaccines and coronavirus vaccines that were published within the current and previous months. Between November 1, 2021, and November 8, 2021, 675 SARS-CoV-2/COVID-19 vaccine publications and 353 coronavirus vaccine publications were published. During the month of October 2021, a total number of 2340 SARS-CoV-2/COVID-19 vaccine publications and 1292 coronavirus vaccine publications were published.

### 3.2 Cov19VaxKB data query and display

**Fig. 2:**
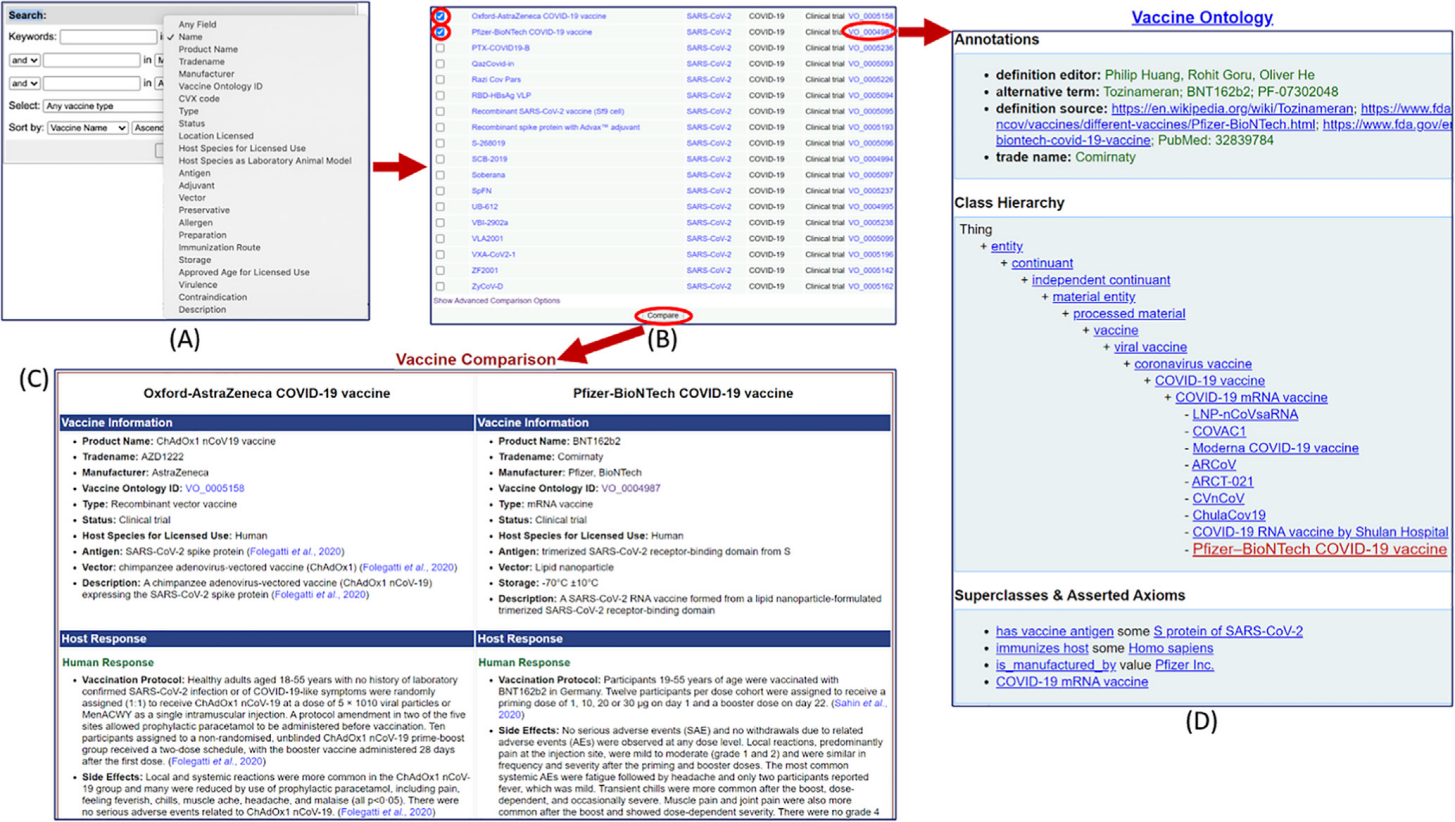
Cov19VaxKB data query. (A) A user can begin by selecting a specific category in the drop-down menu and typing in a keyword that will be used to query vaccines that only contain that keyboard. Up to three different categories can be specified to query a list of COVID-19 vaccines. The query feature also allows the user to sort vaccines according to conditions such as vaccine name or Vaccine Ontology ID. (B) Once the user has clicked “Search,” the query will produce a list of vaccines that satisfy the specified criteria. The user can select one or more of these vaccines and click “Compare” to compare the VIOLIN entries of the desired vaccines. (C) By doing so, the user will be presented with formatted side-by-side lists that contain general vaccine and host response information. General vaccine information contains data such as the product name of the vaccine, vaccine type, and antigen. Host response information contains brief summaries of randomized controlled trial data found in relevant publications. (D) Also, from the list of vaccines generated from the initial query, the user can click on the VO ID link. This will direct the user to a formatted VO vaccine entry in the Ontobee data server.

The Cov19VaxKB provides a user-friendly data query for users to search and compare the COVID-19 vaccine entries stored in the VIOLIN database. The query is located on the homepage of the knowledge base, allowing users to access the feature upon entering the website. A user can begin by selecting a category in the drop-down menu and typing in a keyword that will be used to query vaccines only containing that keyword (Fig. 2A). Up to three different categories can be specified to query a list of COVID-19 vaccines. The query feature also allows the user to sort vaccines according to conditions such as vaccine name or Vaccine Ontology ID. The query generates a filtered list of COVID-19 vaccines with links to their corresponding VIOLIN and VO entries (Fig. 2B). After a queried vaccine list is generated based on the user’s input, multiple vaccines from this list can be chosen to view a formatted side-by-side display of their respective VIOLIN entries, which include product information and host response data (Fig. 2C). Also, from the list of vaccines generated from the initial query, the user can click on a VO ID link to access its formatted VO entry in the Ontobee data server (Fig. 2D).

### 3.3 Analysis of COVID-19 vaccine adverse events

**Fig. 3:**
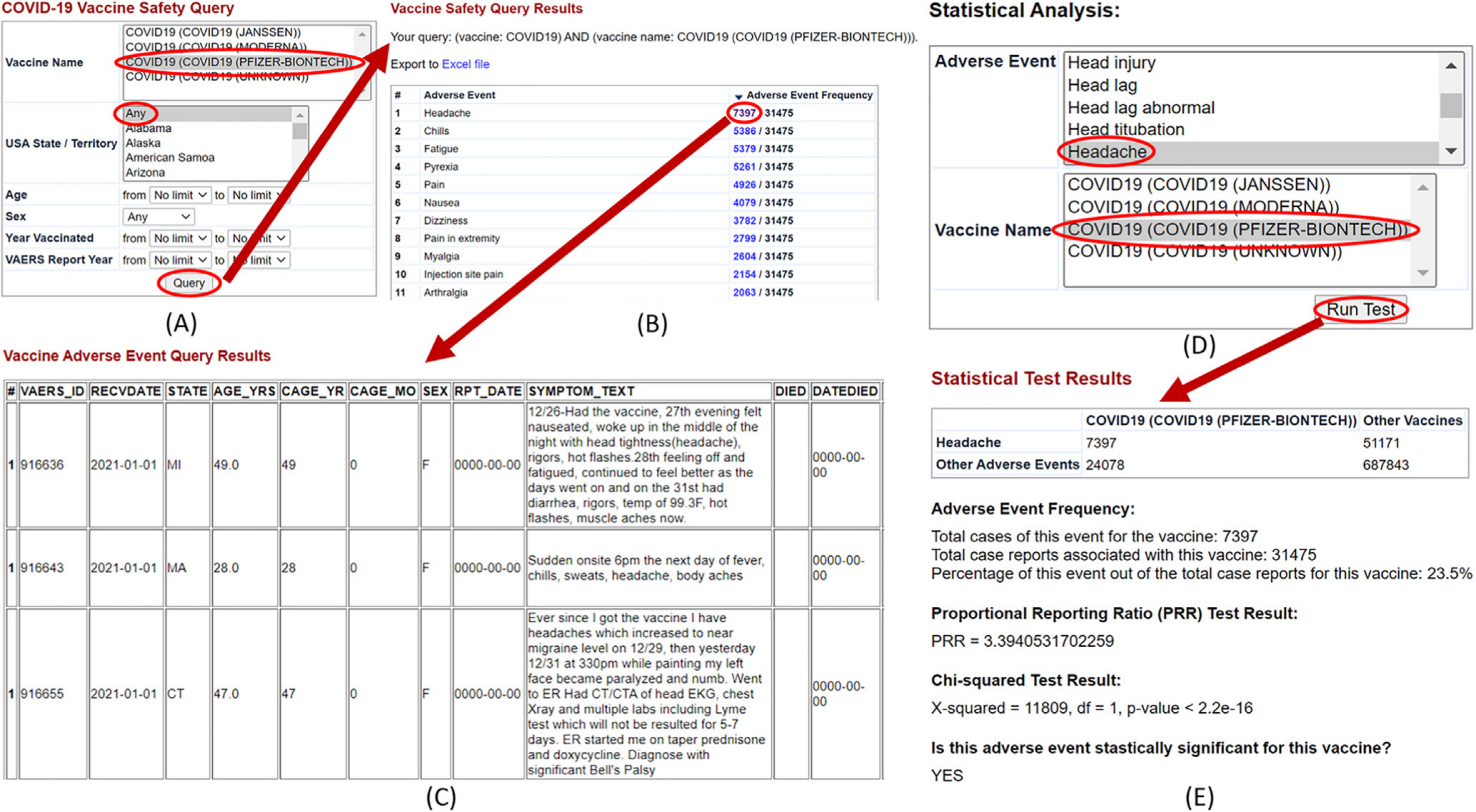
Cov19VaxKB adverse event query and statistical analysis tool. (A) A user can begin by selecting a specific vaccine and specifying various filters including US state/territory, age, sex, year vaccinated, and VAERS report year. (B) After the user clicks “Query,” the query will generate a list of adverse events with their frequency of case reports. (C) The user can click an adverse event frequency to view a detailed table of individual VAERS case reports. (D) To perform statistical analyses, the user can select an adverse event and specify a vaccine of interest. (E) After clicking “Run Test,” the user will be presented with a formatted 2x2 table of AE case report counts and statistical test results, including a Chi-squared analysis, PRR, and statistical significance result.

Users can generate a filtered list of adverse events (AEs) and access a comprehensive table of case reports through the vaccine adverse event query (Fig. 3A-C). As of October 29, 2021, there were a total of 635,799 adverse event reports for all COVID-19 vaccines, among which 44.6% (283,340/635,799) were for the Pfizer-BioNTech COVID-19 vaccine, 46.1% (293,414/635,799) for the Moderna COVID-19 vaccine, 9.1% (57,617/635,799) for the Johnson & Johnson (Janssen) COVID-19 vaccine, and 0.2% (1428/635,799) reports from COVID-19 vaccines for which the manufacturer information is unavailable. On average, the top 10 AEs across the Pfizer-BioNTech, Moderna, and Janssen vaccines were headache (18%), pyrexia (fever) (15%), fatigue (15%), chills (13%), pain (13%), dizziness (10%), nausea (10%), pain in extremity (10%), myalgia (6%), and arthralgia (6%). COVID-19 vaccine-related death report counts included the reports for the following: death, brain death, cardiac death, clinical death, fetal death, maternal death during childbirth, sudden cardiac death, sudden death, stillbirth, completed suicide, agonal death struggle, and drowning. Out of 6673 total death reports, 3035 were for the Pfizer-BioNTech vaccine, 2903 for Moderna, 709 for Janssen, and 26 for COVID-19 vaccines for which the manufacturer information is unavailable.

**Fig. 4:**
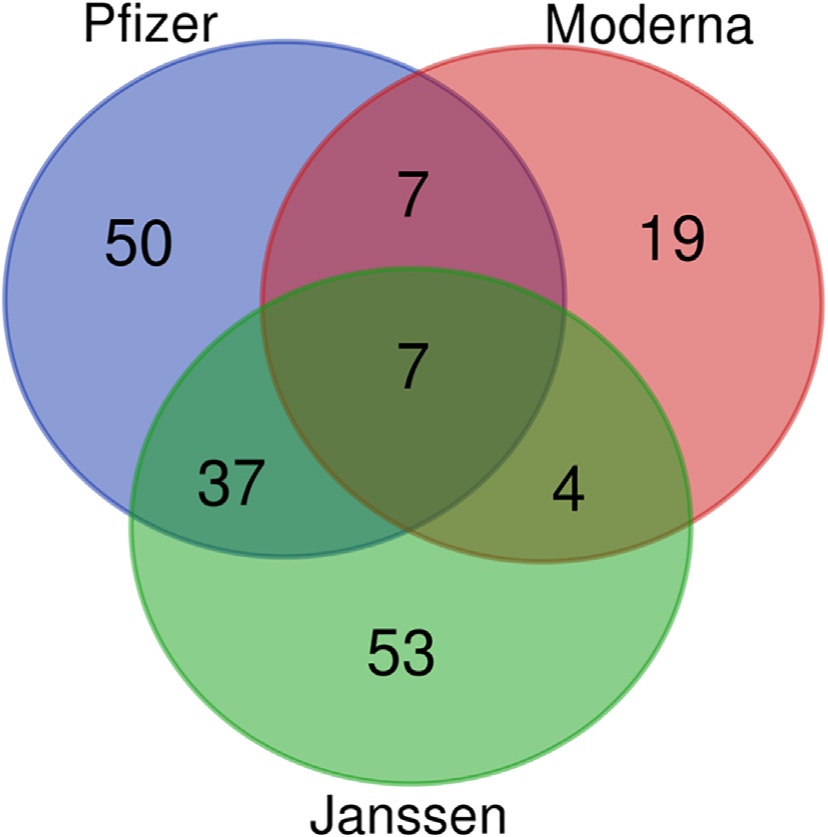
Venn diagram of enriched adverse events. The number and distribution of unique statistically significant AEs for the Pfizer, Janssen, and Moderna COVID-19 vaccines are displayed. The significance cutoff was Chi-squared value > 4, PRR > 2, and number of case reports > 0.2% of total case reports for the specified vaccine.

Statistically significant AEs for each COVID-19 vaccine were identified by the adverse event statistical analysis tool (Fig. 3D-E). The total number of significant AEs was 101 for the Pfizer-BioNTech vaccine, 37 for the Moderna vaccine, and 101 for the Janssen vaccine (Fig. 4). Seven AEs are significant for all 3 vaccines, among which severe AEs include pulmonary embolism and gait inability. Among 7 significant AEs shared between the Pfizer-BioNTech and Moderna vaccines, severe AEs include atrial fibrillation and facial paralysis. Among the 37 significant AEs shared by the Janssen and Pfizer-BioNTech vaccines, more severe ones include seizure and thrombosis. Four non-severe AEs were only significant for the Moderna and Janssen vaccines (Supplemental File 2).

Fifty AEs were only significant in the Pfizer-BioNTech vaccine, including severe AEs such as acute kidney injury and myocardial infarction. Nineteen AEs were only significant for the Moderna vaccine. Most of them were related to the injection site, including rash, pruritus, or skin erythematous. Fifty-three AEs were only significant in the Janssen vaccine, including unresponsiveness to stimuli, loss of consciousness, exertional dyspnea, angiogram pulmonary abnormal, and acute respiratory failure.

### 3.4 Cov19VaxKB vaccine design

The Cov19VaxKB vaccine design tool has identified 24 SARS-CoV-2 vaccine targets as reported in our previous study [15]. The antigen target with the highest Vaxign-ML score is the surface glycoprotein, also known as the spike protein. Unsurprisingly, the surface glycoprotein is the antigen for many existing COVID-19 vaccines, including the Pfizer-BioNTech, Moderna, Johnson & Johnson, and Oxford-AstraZeneca vaccines.

### 3.5 Cov19VaxKB ontological annotation and data sharing

**Fig. 5:**
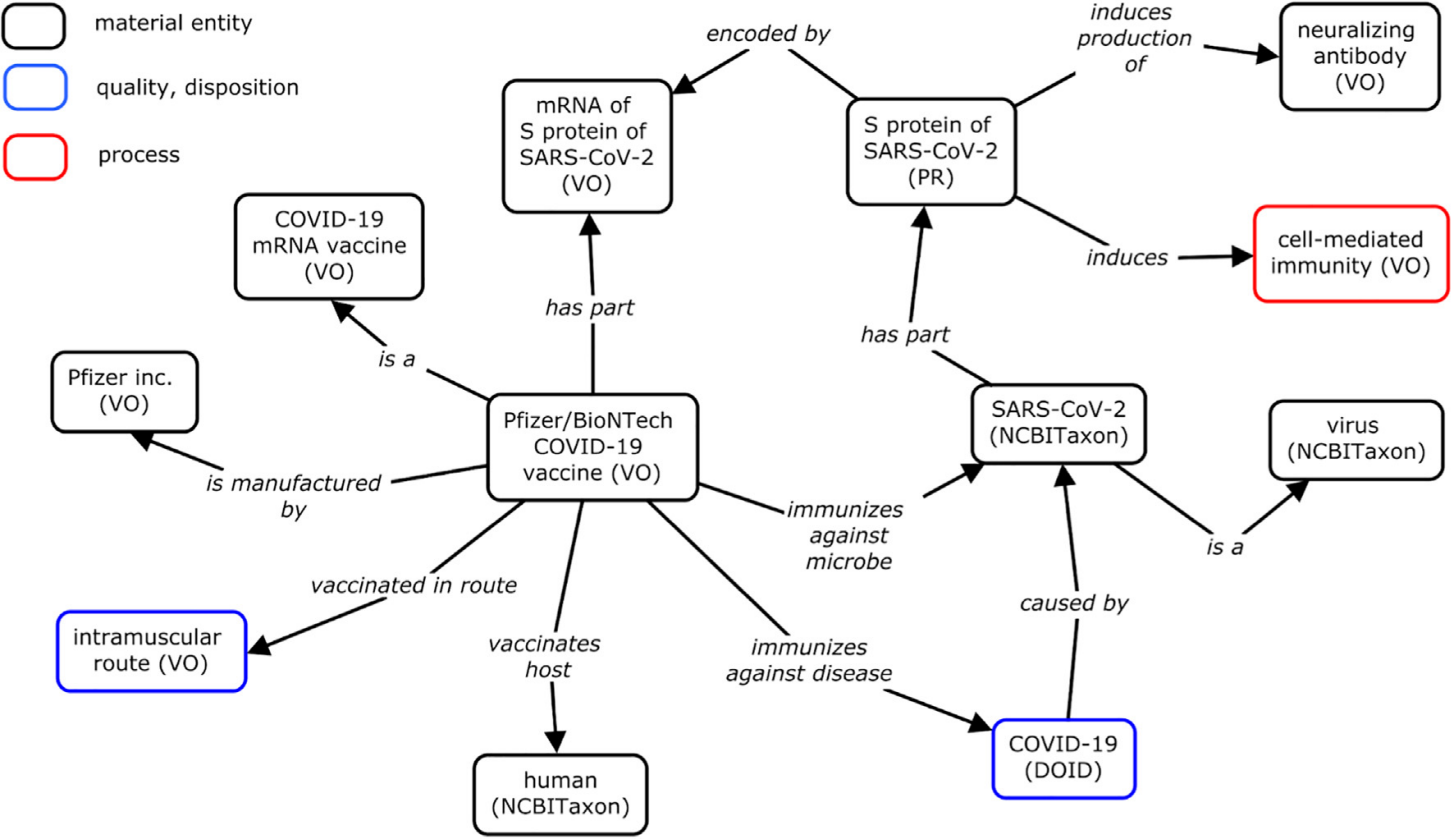
Vaccine Ontology framework for Pfizer/BioNTech COVID-19 vaccine. This design pattern depicts the VO’s logical representations and linkages of different material entities, qualities, dispositions, and processes related to the Pfizer-BioNTech COVID-19 vaccine. The terms in the diagram are either generated in VO or imported from other ontologies.

VO contains entries for all COVID-19 vaccines that have been authorized for public use or that are currently in Phase 1–3 clinical trials. Fig. 5 displays an ontological framework for the Pfizer-BioNTech COVID-19 vaccine. The ontology representation indicates that the “Pfizer-BioNTech COVID-19 vaccine” uses the “mRNA of the S protein of SARS-CoV-2” as its part and immunizes against the virus. The vaccine is administered via the “intramuscular route” and is manufactured by “Pfizer Inc.” The ontology also illustrates that the “S protein” induces “cell-mediated immunity.”

## 4 Discussion

To the best of our knowledge, Cov19VaxKB is the first web-based, publicly available knowledge base that targets the curation and analysis of COVID-19 vaccine information. The knowledge base can be used for a broad range of applications. Comparison of host responses and analysis of adverse events may reveal important statistics and enriched patterns that can promote further studies about COVID-19 vaccine development and safety. Cov19VaxKB’s vaccine lists can be openly accessed to learn about various features of COVID-19 vaccines, including vaccine types, clinical trial information, dates of authorization, vaccine safety information, and relevant publications. Users can predict vaccine targets using the vaccine design tool or utilize the adverse event data analysis feature to determine safety signals for specific COVID-19 vaccines.

The Cov19VaxKB web query enables users to compare product and host response information between various COVID-19 vaccines. Thus, the query can be a powerful tool for analyzing relationships between two or more vaccine properties or attributes. For instance, users can analyze the relationship between immune responses and vaccine type by comparing neutralizing antibody levels and protection rates among vaccines of different types. Vaccine efficacy rates can be compared across different vaccine types to assess any broad differences or similarities in vaccine efficacy.

The adverse event analysis tool can identify enriched adverse events in COVID-19 vaccines that can be analyzed for potential causal relationships in future studies. A statistically significant adverse event for a vaccine represents the enriched association between the vaccination and the adverse event at the population level, but it does not imply that the vaccination induced the adverse event for a specific individual. An adverse event is any undesirable experience that happens after vaccination which may or may not be caused by a vaccine [16]. Overall, existing COVID-19 vaccines have been demonstrated to be safe [17]. Further adverse event analysis is necessary to determine whether instances of adverse events such as death and thrombosis were directly caused by COVID-19 vaccines.

Although statistically significant adverse events occur more frequently for the vaccine of interest compared to other vaccines, these AEs occur at a very low frequency compared to the total number of vaccinations administered. For example, there were only 2736 reported occurrences of pulmonary embolism among the 3 FDA-authorized vaccines, in contrast to a total number of 428,006,540 COVID-19 vaccine doses administered in the United States as of November 6, 2021 [18]. In other words, pulmonary embolism occurred in only 0.0006% of all COVID-19 vaccinations in the US.

In conclusion, Cov19VaxKB provides a timely platform for the curation, sharing, and analysis of COVID-19 vaccine information. In the future, we aim to continue adding new features to the knowledge base to improve the user experience and meet the growing demand for relevant vaccine data analysis. As the volume of COVID-19 vaccine and vaccination data continues to grow, the knowledge base will be a useful and reliable reference for both researchers and the public.

## Declaration of Competing Interest

The authors declare that they have no known competing financial interests or personal relationships that could have appeared to influence the work reported in this paper.
